# Endoscopic evaluation by the Kyoto classification of gastritis combined with serum anti-*Helicobacter pylori* antibody testing reliably risk-stratifies subjects in a population-based gastric cancer screening program

**DOI:** 10.1007/s00535-023-02010-w

**Published:** 2023-06-21

**Authors:** Ryosuke Hirai, Mami Hirai, Motoyuki Otsuka, Toshiharu Mitsuhashi, Yuichi Shimodate, Hirokazu Mouri, Kazuhiro Matsueda, Hiroshi Yamamoto, Motowo Mizuno

**Affiliations:** 1 Department of Gastroenterology and Hepatology, Okayama University Graduate School of Medicine, Dentistry and Pharmaceutical Sciences, 2-5-1, Shikata, Kitaku, Okayama, Okayama 700-8558 Japan; 2 Center for Innovative Clinical Medicine, Okayama University Hospital, 2-5-1, Shikata, Kitaku, Okayama, Okayama 700-8558 Japan; 3 Department of Gastroenterology and Hepatology, Kurashiki Central Hospital, 1-1-1 Miwa, Kurashiki, Okayama 710-8602 Japan

**Keywords:** Cancer screening, Gastric cancer, *Helicobacter pylori*, Gastrointestinal endoscopy, Atrophic gastritis

## Abstract

**Background:**

We previously demonstrated that the Kyoto classification of gastritis was useful for judging the status of *Helicobacter pylori* infection in a population-based screening program, and that adding *H. pylori* antibody test improved its accuracy (UMIN000028629). Here, we tested whether our endoscopic diagnosis of *H. pylori* infection status reliably estimated gastric cancer risk in the program.

**Methods:**

Data were collected from1345 subjects who underwent endoscopic follow-up 4 years after the end of the registration. We analyzed the association of three diagnostic methods of *H. pylori* infection with gastric cancer detection: (1) endoscopic diagnosis based on the Kyoto classification of gastritis; (2) serum diagnosis according to the ABC method (*H. pylori* antibody and pepsinogen I and II); and (3) endoscopic diagnosis together with *H. pylori* antibody test.

**Results:**

During the follow-up, 19 cases of gastric cancer were detected. By Kaplan–Meier analysis, the detection rates of cancer were significantly higher in the past or current *H. pylori* infection groups than in the never-infected group with all 3 methods. By the Cox proportional hazards model, the hazard ratio for cancer detection was highest in evaluation with the combined endoscopic diagnosis and the antibody test (method 3; hazard ratio 22.6, 95% confidence interval 2.99–171) among the three methods (the endoscopic diagnosis (method 1); 11.3, 2.58–49.8, and the ABC method (method 2); 7.52, 2.49–22.7).

**Conclusions:**

Endoscopic evaluation of *H. pylori* status with the Kyoto classification of gastritis, especially combined with serum anti-*Helicobacter pylori* antibody testing, reliably risk-stratified subjects in a population-based gastric cancer screening program.

## Introduction

In the population-based gastric cancer screening program in Japan, esophagogastroduodenoscopy (EGD) has been an option since 2016; in the program, gastric cancer screening has consisted of EGD or radiography every 2 years in all people above 50 years of age. A major drawback of the program is that *Helicobacter pylori* infection status, which is a major risk factor for gastric cancer [1, 2], is not acknowledged. In Japan, the rate of *H. pylori* infection is declining [3]; thus, the program involving all people above a certain age has become a waste of resources. Rather, a screening system in which *H. pylori* infection status is appreciated to stratify the gastric cancer risk and achieve more efficient screening has been needed.

Thus, in 2017, we started a population-based endoscopic gastric cancer screening program with consideration of *H. pylori* infection based on the Kyoto classification of gastritis, which has been found useful for evaluating *H. pylori* infection status endoscopically [4, 5], in Kurashiki City. In our prospective case-registration study (UMIN000028629) to evaluate feasibility of this attempt, we learned that endoscopic evaluation of gastritis is useful for judging *H. pylori* infection status, and that adding *H. pylori* serum antibody test improved its accuracy [6]. Since then, we have followed the participants endoscopically for nearly 5 years. In the present study, we aimed to determine whether endoscopic diagnosis of *H. pylori* infection status, with concomitant *H. pylori* antibody testing, reliably defined gastric cancer risk of the subjects in the population-based screening program.

## Materials and methods

### Study design and subjects

**Fig. 1:**
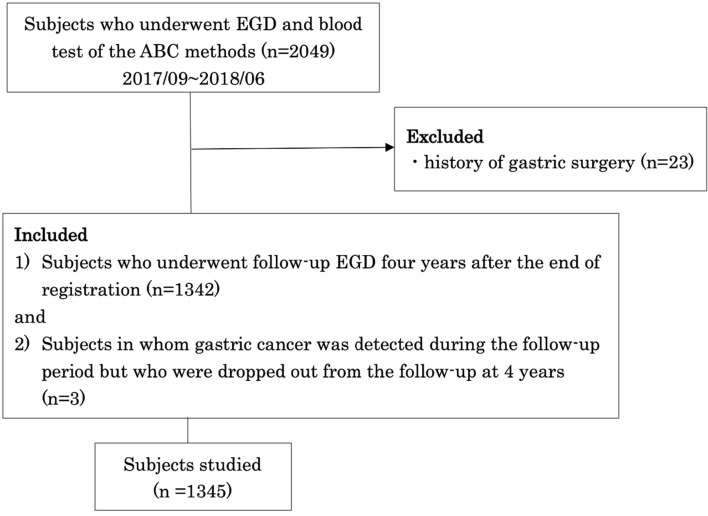
Flowchart of subject selection. Six hundred eighty-one patients who did not met the inclusion criteria were excluded

We recruited subjects from 2049 participants of the prospective case-registration study (UMIN000028629), who had undergone EGD for gastric cancer screening in Kurashiki Central Hospital Preventive Healthcare Plaza affiliated to Kurashiki Central Hospital from September 2017 to June 2018. All subjects had undergone serum tests and endoscopic evaluation on the same day. Based on the endoscopic or the serum diagnosis of *H. pylori* infection, the participants with past or current infection were asked to have annual endoscopic follow-up, and those never infected with *H. pylori* were asked to have biennial endoscopic follow-up. In cases diagnosed as active gastritis, eradication treatment was recommended. Among the 2049 participants, 23 participants with a history of gastric surgery were excluded. Of the remaining 2026 participants, 1342 subjects who underwent follow-up EGD four years after the end of registration and 3 subjects in whom gastric cancer was detected during the follow-up period but who were dropped out from the follow-up at 4 years (1345 subjects in total), were studied (Fig. 1). Subjects’ age, sex, family history of gastric cancer, history of examination for or eradication of *H. pylori*, and history of gastric or abdominal surgery, were collected. Gastric cancer was basically classified according to Lauren as intestinal type or diffuse type [7]. Gastric-type tumor such as foveolar-type dysplasia was separately categorized according to the WHO Classification of Tumours [8].

The study was conducted according to the guidelines of the Declaration of Helsinki. The study was approved by the institutional review board of the Kurashiki Central Hospital in March 2018 (approval number: 2540) and was registered with UMIN Clinical Trials Registry (UMIN000028629). The objective of the study was explained to all subjects before their participation, and a written informed consent was obtained from each subject.

### Endoscopic evaluation of *H. pylori* infection

In the first year, EGD was performed with an EG-L580NW scope and light source LASEREO (FUJIFILM Medical Co., Ltd., Tokyo, Japan). At endoscopic examination, we evaluated the degree of atrophy and *H. pylori* infection status [6]. Gastric mucosal atrophy was classified by degree into grades C-0 (none), C-1, C-2, C-3, O-1, O-2, and O-3 according to the endoscopic–atrophic-border scale described by Kimura and Takemoto [9–11]. Regarding *H. pylori* infection status, findings described in the Kyoto classification of gastritis [5] such as presence or absence of regularly arranged collecting venules, enlarged gastric folds, nodularity, diffuse and/or spotty redness, and map-like or patchy redness of the gastric mucosa were evaluated. Based on these findings, gastric mucosa was endoscopically classified into non-gastritis (looking like never infected with *H. pylori*), active gastritis (current *H. pylori* infection), inactive gastritis (past infection), or undefined (equivocal or status of gastritis difficult to judge). The main diagnostic criteria were gastric mucosal atrophy with diffuse and/or spotty redness for active gastritis; atrophy with map-like redness and/or patchy redness for inactive gastritis; and regularly arranged collecting venules in the lesser curvature of the gastric angle for non-gastritis [6].

### Serum test for *H. pylori* infection

For comparison of the diagnosis of *H. pylori* infection, a combined serum test of anti-*H. pylori* antibody and pepsinogen (PG) I and II, the ABC method [12–14], was used. According to serum antibody to *H. pylori* and pepsinogen I and II values, *H. pylori* infection status and grade of gastric atrophy were classified into Group A–D: A, *H. pylori* antibody (−) and gastric atrophy (−); B, antibody (+) but atrophy (−); C, antibody (+) and atrophy (+); D, antibody (−) and atrophy (+). As reported [6], the serum antibody to *H. pylori* was measured with an enzyme immunoassay method (E-plate, Eiken Chemical, Tokyo, Japan), and a cutoff value of ≥ 3 U/mL was used to reduce false-negative results [15]. PGI and II were measured with the chemiluminescent enzyme immunoassay method (Fujirebio, Tokyo, Japan), and PG I of ≤ 70 ng/mL and PG I/II ratio of ≤ 3.0 were considered positive for PG test [16].

### Outcomes

The primary outcome was the association of (1) the endoscopic diagnosis of *H. pylori* infection based on the Kyoto classification of gastritis, (2) serum diagnosis according to the ABC method, and (3) the endoscopic diagnosis together with serum *H. pylori* antibody test, with the gastric cancer detection during the study period. In a sub-analysis, association of the gastric atrophy score of the Kyoto classification of gastritis; score 0 (no atrophy ~ C-1 of the endoscopic atrophy scale described by Kimura and Takemoto [9–11]), score 1 (C-2 ~ C-3), and score 2 (O-1 ~ O-3), with the gastric cancer detection was examined.

### Statistical analysis

We assessed the factors associated with the detection of gastric cancer by Cox’s proportional-hazards models. Statistical differences were calculated with the Mann–Whitney *U* test, the *χ*^2^ test, and Fisher’s exact test. Hazard ratios and 95% confidence intervals (CIs) were calculated by Cox proportional hazard model, and *p* < 0.05 was considered statistically significant. Survival curves were constructed with the Kaplan–Meier method, and significant differences between curves were tested with the log-rank test. For statistical analysis, we used EZR (Saitama Medical Center, Jichi Medical University, Saitama, Japan), which is a graphical user interface for R (the R Foundation for Statistical Computing, Vienna, Austria).

## Results

Characteristics of the 1345 study subjects are presented in Table 1. The median age was 56 years, and 54.2% were male. One hundred ninety-two subjects (14.3%) had a history of *H. pylori* eradication therapy. The endoscopic findings of gastritis according to the Kyoto classification of gastritis and the results of the ABC method are presented in Table 1, and the relationship between them is summarized in Table 2. The endoscopic diagnosis was non-gastritis in 872 (64.9%) subjects and active or inactive gastritis in 471 (35.0%) subjects. The results of the ABC method were group A, *n* = 930 (69.1%); group B ~ D, *n* = 415 (B, *n* = 324; C, *n* = 80; and D, *n* = 11); the prevalence of current or past *H. pylori* infection according to the ABC methods was 30.9% (415/1345) in the study subjects. With the ABC method as a reference standard for *H. pylori* infection, the false-negative rate of the endoscopic judgment for *H. pylori* infection was 6.5% (27/415, 95% CI 4.5–9.3%). In contrast, the false-negative rate of the ABC method with endoscopic diagnosis of gastritis as a reference was surprisingly high and reached 17.8% (84/471, 95% CI 14.6–21.6%). With a positive result of either endoscopic diagnosis or the ABC method as a reference standard for *H. pylori* infection (*n* = 499), the false-negative rates of the endoscopic judgment for *H. pylori* infection and the ABC method were 5.4% (27/499, 95% CI 3.7–7.8%) and 16.8% (84/499, 95% CI 13.8–20.4%), respectively.

**Table 1:** Characteristics of study subjects (*n* = 1345)

Age (years)	56.0 ± 11.3
Male	729 (54.2%)
*H. pylori* eradication therapy before enrollment	192 (14.3%)
Kyoto classification of gastritis^a^	
Atrophy	471 (35.0%)
Intestinal metaplasia	156 (11.6%)
Diffuse and/or spotty redness	327 (24.3%)
Mucosal swelling and/or enlarged fold	256 (19.0%)
Nodularity	22 (1.6%)
RAC on angular region and antrum	853 (63.4%)
Endoscopic diagnosis of gastritis^b^	
Active gastritis	247 (18.4%)
Inactive gastritis	224 (16.6%)
Non-gastritis	872 (64.9%)
Undefined	2 (0.1%)
ABC method^c^	
A	930 (69.1%)
B	324 (24.1%)
C	80 (6.0%)
D	11 (0.8%)

*RAC* regular arrangement of collecting venules, *PG* pepsinogen

^a^Positive criteria of each item of the Kyoto classification of gastritis: score 1 or 2 for Atrophy, Intestinal metaplasia, and Diffuse and/or spotty redness; score 1 for Mucosal swelling and/or enlarged fold and Nodularity [5]; and score 0 for RAC on angular region and antrum

^b^The main diagnostic criteria; gastric mucosal atrophy with diffuse and/or spotty redness for active gastritis, atrophy with map-like redness and/or patchy redness for inactive gastritis, and regularly arranged collecting venules in the lesser curvature of the gastric angle for non-gastritis

^c^The ABC method: Group A, *Helicobacter pylori* antibody (−) PG (−); Group B, *H. pylori* antibody (+) PG (–); group C, *H. pylori* antibody (+) PG (+); and group D, *H. pylori* antibody (−) PG (+). PG, positive if PGI and PG I/II ratios were ≤ 70 ng/mL and ≤ 3.0, respectively

**Table 2:** Relationship between the diagnosis of *H. pylori* infection status according to the Kyoto classification of gastritis and the ABC method

Endoscopic findings	ABC method	
	Group A (930, 69.1%)	Groups B–D (415, 30.9%)
Non-gastritis (872, 64.9%)	845	27^a^
Active or inactive gastritis (471, 35.0%)	84^b^	387
Undefined (2, 0.1%)	1	1

^a^With the ABC methods as a reference standard for *Helicobacter pylori* infection, the false-negative rate of the endoscopic judgment for *H. pylori* infection was 6.5% (27/415, 95% CI 4.5–9.3%)

^b^The false-negative rate of the ABC method with endoscopic judgment of *H. pylori* status as a reference was 17.8% (84/471, 14.6–21.6%)

During the study period, 19 cases of gastric cancer were detected. Four patients had gastric cancer by EGD at the enrollment. Characteristics of the 19 cancers are presented in Table 3. Histologically, 13 of the cancers were intestinal type, 4 were diffuse type according to Lauren [7], and 2 were gastric type (foveolar-type dysplasia) which were separately categorized and were regarded as Tis gastric cancer according to the WHO Classification of Tumours [8]. Most gastric cancers were in early TNM stages (stage 0, *n* = 16; stage IA, *n* = 2), but one case had advanced gastric cancer with liver metastasis (cStage IVB) [17]. Endoscopically, most gastric cancers were detected in the background of active or inactive gastritis, and gastric-type tumors were detected in two subjects with non-gastritis. In the ABC method, gastric cancer was detected in 4 and 15 subjects with Group A and B–D, respectively.

**Table 3:** Characteristics of 19 gastric cancers

	*N* = 19
Histological type	
Intestinal type^a^	13 (68.4%)
Diffuse type	4 (21.1%)
Gastric type^b^	2 (10.5%)
TNM stage	
0 (TisN0M0)	16 (84.2%)
IA (T1N0M0)	2 (10.5%)
IVB (cT3N1M1)	1 (5.3%)
Location in stomach	
Upper-third	3 (15.8%)
Middle-third	4 (21.1%)
Lower-third	12 (63.1%)
Endoscopic diagnosis of gastritis	
Non-gastritis	2 (15.8%)
Active or inactive gastritis	17 (84.2%)
ABC method	
A	4 (21.1%)
B–D	15 (78.9%)

^a^Gastric cancer was classified according to Lauren as intestinal type or diffuse type [7]

^b^Gastric-type tumor; 2 tumors of foveolar-type dysplasia in this study were separately categorized according to the WHO Classification of Tumours [8] and were regarded as Tis gastric cancer

**Fig. 2:**
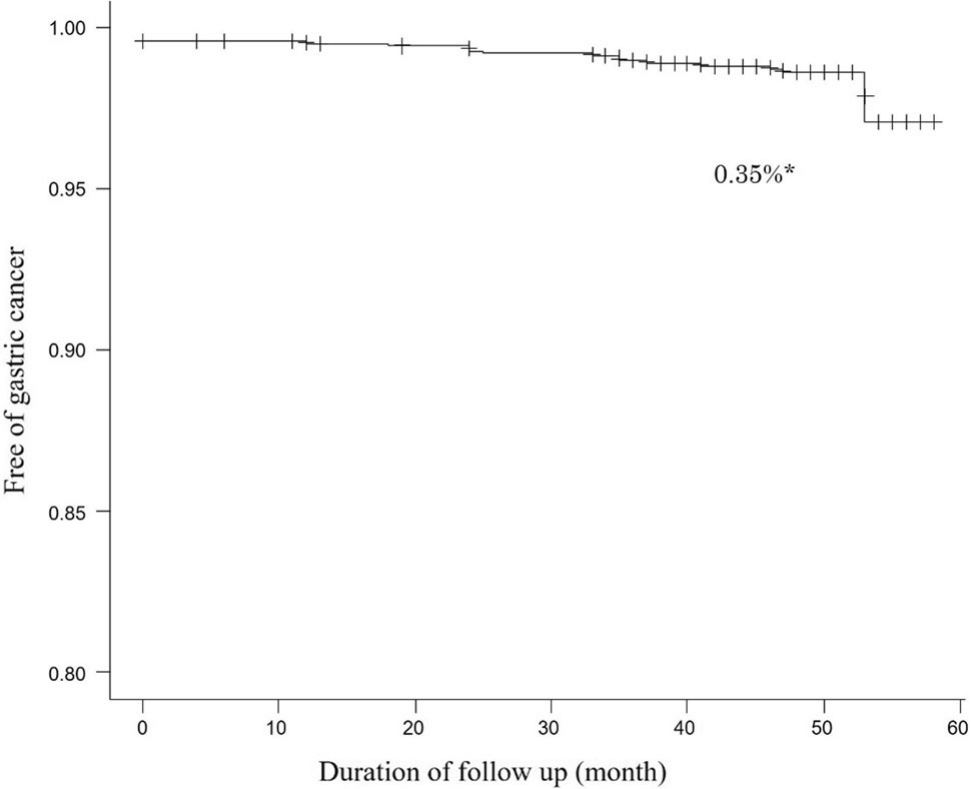
Kaplan–Meier analysis of the proportion of patients who remained free of gastric cancer. *The annual rate of detecting gastric cancer calculated by Kaplan–Meier analysis

**Fig. 3:**
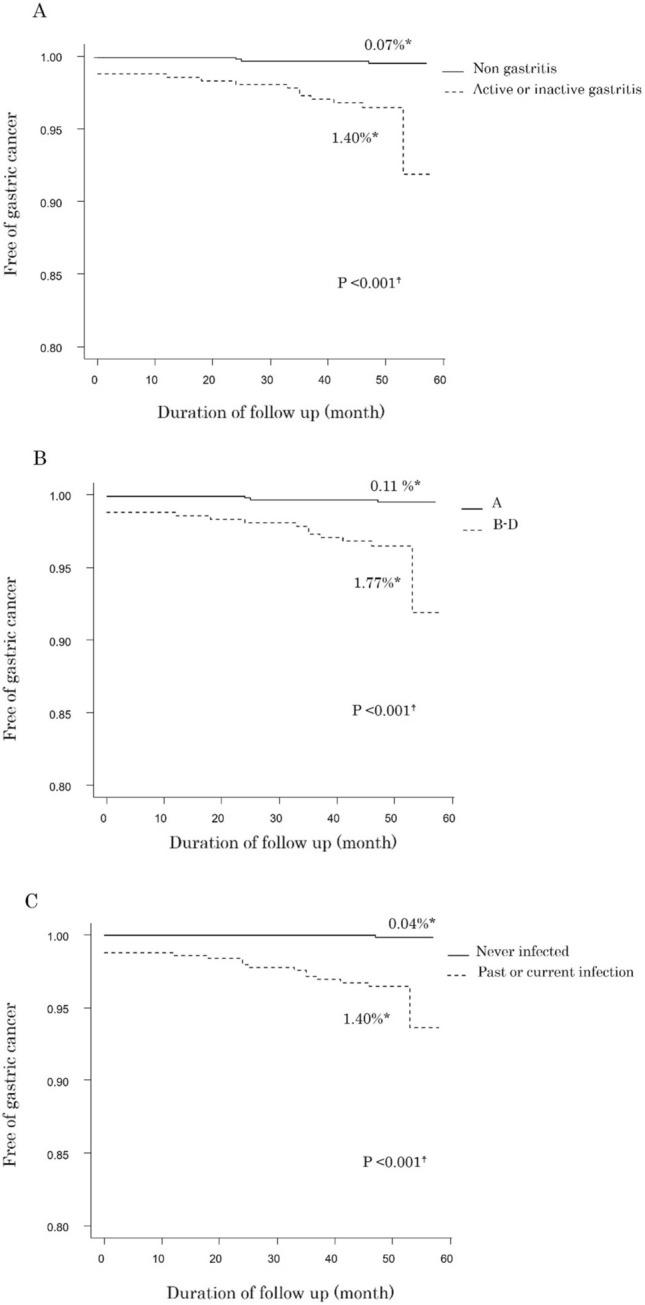
Kaplan–Meier analysis of the proportion of patients who remained free of gastric cancer according to *H. pylori* infection status at the time of enrollment. **A** Curves according to *H. pylori* infection status based on the endoscopic diagnosis with Kyoto classification of gastritis. **B** Curves according to serologically judged *H. pylori* infection status (the ABC method). **C** Curves according to combined evaluation of *H. pylori* infection status with the endoscopic diagnosis and serum *H. pylori* antibody. *The annual risk of detecting gastric cancer calculated by Kaplan–Meier analysis. †Statistical significance between curves tested by log-rank test

By Kaplan–Meier analysis, the rate of gastric cancer detection was 0.35% per year (Fig. 2). Kaplan–Meier curves of detection of gastric cancer according to each *H. pylori* infection status based on the endoscopic diagnosis of gastritis and the ABC method are presented in Fig. 3. With both endoscopically and serologically judged *H. pylori* infection status, the detection rate of gastric cancer was significantly higher in the groups judged to have past or current *H. pylori* infection than in the never-infected groups (Fig. 3A, B). In our previous work, we found that false-negative judgment of *H. pylori* infection with the endoscopic diagnosis could occur, especially in patients with mild gastric atrophy. To avoid the limitation, we proposed adding *H. pylori* antibody test to the endoscopic diagnosis. With this combined evaluation of *H. pylori* infection status, detection of gastric cancer was also significantly higher in the current or past-infected group than in the never-infected group (Fig. 3C). To rank superiority of each method for risk prediction of gastric cancer, we calculated the hazard ratio for gastric cancer detection with the Cox proportional hazards model (Table 4). The hazard ratio calculated with age and sex as covariates was highest in the combined evaluation with the endoscopic diagnosis and serum anti-*H. pylori* antibody (hazard ratio 22.6, 95% confidence interval 2.99–171, *p* = 0.0025) among the 3 methods (the endoscopic diagnosis alone, 11.3, 2.58–49.8, *p* = 0.0013 and the ABC method, 7.52, 2.49–22.7, *p* = 0.0004). In the sub-analysis according to the gastric atrophy score of the Kyoto classification of gastritis, the hazard ratios for gastric cancer detection were score 0, 0.07 (95% confidence interval 0.01–0.33); score 1, 0.23 (0.03–1.76); and score 2, 24.7 (6.83–89.0).

**Table 4:** Rank analysis of each method of diagnosis of *H. pylori* infection status for risk stratification of gastric cancer detection with Cox’s proportional hazards model

	Hazard ratio	95% CI	*p* value
Endoscopic diagnosis of gastritis (inactive or active gastritis)	11.3	2.58–49.8	0.0013
ABC method (B–D)	7.52	2.49–22.7	0.0004
Combined diagnosis (past or current infection)	22.6	2.99–171	0.0025

*95% CI* 95% confidence interval

## Discussion

In this study, we found that the Kyoto classification of gastritis, especially with *H. pylori* antibody testing added, reliably predicted gastric cancer risk of the subjects in a population-based gastric cancer screening program. The results of present study advanced our previous work [6], in which we showed the feasibility of endoscopic diagnosis of *H. pylori* infection according to the Kyoto classification of gastritis in the screening program.

There have been several reports to improve efficacy of the gastric cancer screening program. In studies from Japan and South Korea, the interval between examinations and the target age group have been discussed [18–20]. A microsimulation modeling study with a virtually created population model showed the cost-effectiveness of gastric cancer screening in multiple scenarios in relation to the target age and screening intervals [21]. However, these studies ignored the most important risk factor for gastric cancer, i.e., *H. pylori* infection. Our findings indicate that endoscopic diagnosis, especially with *H. pylori* antibody testing, enables gastric cancer screening with *H. pylori* status accounted. Thus, the target age and the examination interval should be re-examined in the gastric cancer screening program where the most important gastric cancer risk is considered.

In addition to the stratification of gastric cancer risk, our endoscopic screening program provides another important benefit. In the present study performed in our institution, diagnosis of inactive or active gastritis was made separately. However, in the population-based endoscopic gastric cancer screening program in Kurashiki City, participants are screened for presence of the gastritis, but whether the gastritis, if present, is inactive or active is not asked because its judgement is occasionally difficult. Thus, persons with inactive gastritis or active gastritis, i.e., possible past or current *H. pylori* infection are advised to have further examination for *H. pylori* infection status. For those with past infection judged by further examination, careful surveillance could be offered, and those with current infection could receive eradication therapy, with the chance of reduced gastric cancer risk. Since we first reported in 2005 the preventive effect of *H. pylori* eradication against gastric cancer in a prospective cohort study [22], other studies, including randomized control studies, have confirmed that eradication of *H. pylori* reduces gastric cancer risk [23–27]. The population-based gastric cancer screening program with endoscopic diagnosis of *H. pylori* infection status can detect gastric cancer reliably by judging subjects’ gastric cancer risk with the chance to reduce the risk itself.

The rate of gastric cancer detection, 0.35% per year, in the present study was the same as in our previous work [28]. In that work, we studied long-term gastric cancer risk in subjects after eradication of *H. pylori* who still had some gastric cancer risk although lower than in those having current *H. pylori* infection. Given that 65 to 69% of the subjects in the present study were judged to be never infected with *H. pylori* (Table 2), who are supposed to be at very low risk of gastric cancer, the rate of 0.35% in the present study appears high. The high rate was most likely due to inclusion of 4 cancers detected with EGD at enrollment in the present study, whereas subjects with gastric cancer at enrollment were excluded in the previous work.

The risk of gastric cancer is closely associated with grade of background gastric atrophy [29]. In the sub-analysis of the present study, the gastric atrophy score of the Kyoto classification of gastritis was also a strong predictor of gastric cancer. When endoscopists involved in the population-based endoscopic gastric cancer screening program get used to the scoring system, adding the atrophy score in the report items in addition to the *H. pylori* infection status may further improve the risk stratification of gastric cancer.

Limitations of our study are (1) for the diagnosis of *H. pylori* infection, we used the endoscopic method and the ABC method, but the sensitivity by either method was not ideal. In determining *H. pylori* status, we used the ABC method as a reference standard for evaluating the endoscopic diagnosis, and endoscopic diagnosis as a reference standard for comparison with the ABC method: the dependability of both methods, however, appears limited. Other methods for determining *H. pylori* infection status, e.g., bacterial culture, histological evaluation of gastritis, stool antigen test, and urease-based tests, may be more reliable. However, gastric biopsy for this purpose is not recommended in population-based screening programs, and urease-based tests or stool antigen quantification detect only current infection. Thus, we arbitrarily calculated the false-negative rates by using endoscopic diagnosis or the result of the ABC method as a reference standard. Nonetheless, our results revealed that endoscopic diagnosis, especially together with the serum antibody test, strongly predicted gastric cancer risk, indicating that our methods are useful in practice. (2) This study is a prospective cohort study, but it was conducted at a single center only. In population-based gastric cancer screening programs, endoscopists with various levels of skill and knowledge for endoscopic diagnosis according to the Kyoto classification of gastritis in private clinics are involved; whether our findings are applicable in such situations needs to be tested.

## Conclusion

In a population-based gastric cancer screening program, endoscopic *H. pylori* status evaluation with the Kyoto classification of gastritis, especially when combined with serum *H. pylori* antibody testing, reliably risk-stratified the participants. *H. pylori* -infected persons, whether with current or past infection, identified through this approach, will be offered careful surveillance. Persons with current infection can have eradication therapy, which should reduce their gastric cancer risk.
